# Lenalidomide-Associated ITP

**DOI:** 10.1155/2011/638020

**Published:** 2011-07-17

**Authors:** Christina I. Herold, Cristina Gasparetto, Gowthami M. Arepally

**Affiliations:** ^1^Division of Hematology/Oncology, Beth Israel Deaconess Medical Center, Boston, MA 02215, USA; ^2^Stem Cell Therapy, Duke University Medical Center, Durham, NC 27710, USA; ^3^Division of Hematology, Duke University Medical Center, Durham, NC 27710, USA

## Abstract

Lenalidomide is a potent immunomodulatory agent being used increasingly for treatment of hematologic malignancies including multiple myeloma and myelodysplasia. The common toxicities of lenalidomide, including dose-limiting myelosuppression, are well described. However, the immunomodulatory properties of lenalidomide may give rise to unexpected autoimmune complications. Herein, we describe a case of immune thrombocytopenic purpura (ITP) associated with use of lenalidomide.

## 1. Introduction

Lenalidomide (CC-5013, REVLIMID; Celgene Corp., NJ, USA) is a potent second-generation derivative of thalidomide approved in the United States for treatment of multiple myeloma and low- to intermediate-risk myelodysplastic syndrome (MDS) caused by deletion of chromosome 5q (5q-syndrome). Lenalidomide and thalidomide belong to the IMiD (immunomodulatory drug) class of therapeutic agents and show antitumor efficacy through a variety of mechanisms, including inhibition of cytokines signaling and angiogenesis, tumor-stromal cell interactions, and induction of tumor cell apoptosis [1].

The immunomodulatory properties of these drugs, in particular, thalidomide, have been also successfully utilized for treatment of nonmalignant diseases, including cutaneous and systemic inflammatory disorders, such as erythema nodosum leprosum [2], Crohn's disease [3], and systemic lupus erythematosus [4]. While in most cases IMiDs appear to down regulate immune responses, these agents can also perturb immune balance and give rise to autoimmunity. For example, alpha interferon, an IMiD used for treatment of both inflammatory and malignant conditions, is known to cause a variety of autoimmune conditions including hemolytic anemia, ITP (immune thrombocytopenic purpura), and thyroiditis [5]. Recent reports of autoimmune thyroiditis with thalidomide and lenalidomide suggest that these IMiDs also have the potential to dysregulate immunity [6, 7]. In this paper, we describe a case of acute ITP likely caused by treatment with lenalidomide therapy.

## 2. Case Presentation

**Figure 1:**
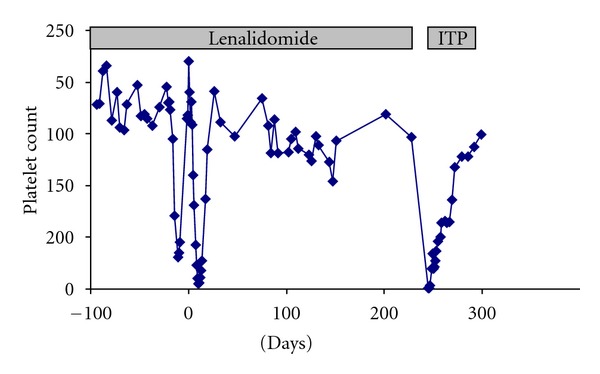
Trend of platelet counts, day 0 represents autologous stem cell transplantation.

The patient is a 27-year-old male with no past medical history who was diagnosed with multiple myeloma and multiple osseous plasmacytomas in February 2008 on presentation with hip pain and a palpable rib mass. Laboratory studies at time of myeloma diagnosis are shown in Table 1. The patient was initially treated for his myeloma with radiation therapy to the right pelvis. He then received four cycles of bortezomib (1.3 mg/m^2^ on days 1, 4, 8, 11 of 3-week schedule), lenalidomide (15 mg daily for 14 days), and dexamethasone (40 mg po weekly) from April through June of 2008; platelet counts during this treatment ranged from 121,000 to 216,000 (Figure 1).

The patient received an autologous peripheral blood stem cell transplantation in July 2008 with a priming regimen consisting of cyclophosphamide (4 g/m^2^ on day-21) and melphalan (200 mg/m^2^ on day-1). His outpatient posttransplantation course was notable for severe thrombocytopenia that was unresponsive to platelet transfusions for approximately two weeks (Figure 1). Platelet counts recovered to 135,000 by day +19 following transplant. From October through December of 2008, the patient received consolidation chemotherapy consisting of four additional cycles of bortezomib (1.3 mg/m^2^ on days 1, 4, 8, 11 of 3-week schedule), lenalidomide (15 mg daily for 14 days), and dexamethasone (40 mg po weekly). Following consolidation, he was maintained on single-agent lenalidomide (25 mg daily).

In January 2009, the patient described new onset of alopecia involving his facial, leg, and chest hair. In early March 2009, the patient developed new leukopenia (WBC 3.1), but other cell counts including hemoglobin, hematocrit, and platelet counts remained stable (Table 1). Due to concern for progressive leukopenia, the patient's lenalidomide was discontinued on March 10th, 2009. Approximately 17 days after discontinuing lenalidomide, the patient developed new petechiae on his lower legs and was found to have a platelet count of 1,000.

**Figure 2:**
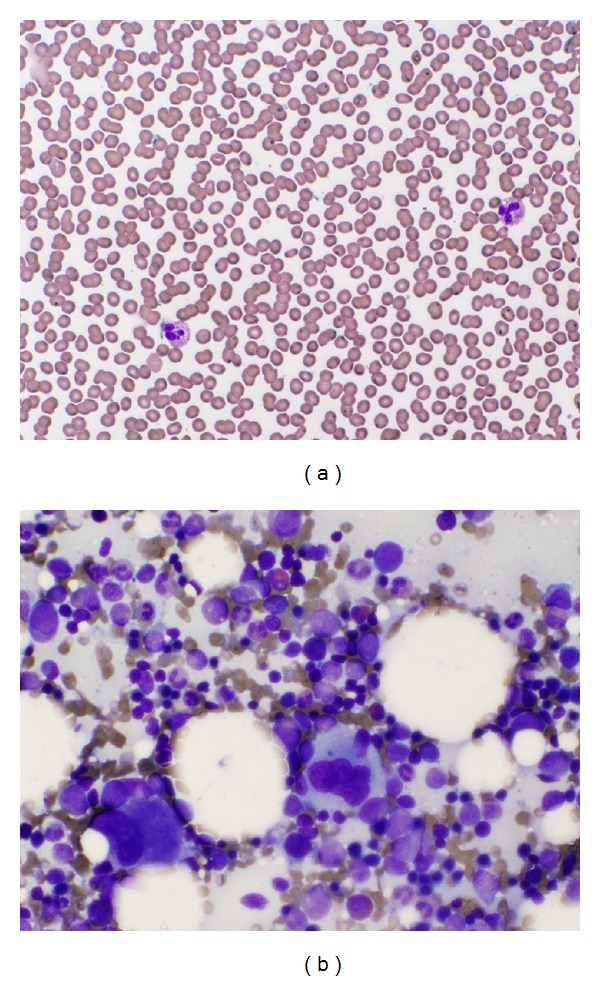
Peripheral blood film (a) and bone marrow aspirate (b) at ITP diagnosis, both 40x magnification.

On presentation with acute thrombocytopenia, the patient reported no recent travel, no viral illnesses, and no new medications. Laboratory studies obtained at time of new thrombocytopenia are shown in Table 1. Peripheral blood film confirmed absence of platelets and no evidence of schistocytes or dysplasia (Table 1 and Figure 2(a)). A bone marrow biopsy revealed normocellular bone marrow with trilineage hematopoiesis and adequate megakaryocytes (Table 1 and Figure 2(b)). There was no increase in plasma cells. Drug-dependent antibodies to lenalidomide were negative. Restaging studies performed in March 2009, 10 days prior to presentation with ITP, included PET scan which revealed innumerable lytic lesions within the axial skeleton but no significant interval changes compared to scans from August 2008.

The patient was diagnosed with acute ITP and was initially treated with high-dose dexamethasone and intravenous immunglobulins. As shown in Figure 1, platelet counts steadily increased on tapering of steroid therapy.

## 3. Discussion

We report a case of acute thrombocytopenia occurring in the context of maintenance lenalidomide therapy for treatment of multiple myeloma. The differential diagnosis for acute thrombocytopenia in adults is broad and includes viral or bacterial illness, autoimmune illness including ITP, thrombotic thrombocytopenic purpura (TTP), and medications. This differential diagnosis is further expanded in this patient due to his underlying plasma cell disorder, recent autologous bone marrow transplant with alkylator conditioning therapy, and history of multiple transfusions. 

Of the various considerations in the patient's differential diagnosis, we were able to exclude several disease processes based on clinical presentation and laboratory evaluation. The patient gave no history of recent viral or bacterial illness or exposure to new drugs, either therapeutic or recreational. Additionally, his laboratory evaluation did not reveal new viral illness {negative serologies for human immunodeficiency virus (HIV), hepatitis or Epstein-Barr virus (EBV)}, autoimmune disease (negative ANA, Coombs), TTP (normal LDH, hemoglobin and peripheral blood film), or posttransfusion purpura (no recent transfusions and negative serologies for PLA1). Underlying myelodysplasia (MDS) from alkylator therapy was also excluded based on timing, therapy-related MDS typically occuring 5–7 years after exposure, as well as normal bone marrow findings and normal cytogenetics. The rapid decline of platelet counts over a two-week period coupled with findings of normal to increased megakaryocytes on bone marrow examination suggested that thrombocytopenia in this patient was likely immune mediated.

Immune thrombocytopenia, or ITP, in adults is a diagnosis of exclusion. ITP frequently accompanies lymphoproliferative diseases, such as chronic lymphocytic leukemia (CLL) and non-Hodgkin's lymphoma, but is rarely seen in patients with plasma cell dyscrasias. Only five cases of myeloma-related ITP have been reported in the literature [8, 9], with four of five cases having evidence of myeloma at ITP presentation and all five cases manifesting paraproteinemia at time of thrombocytopenia. While it is possible that our patient's ITP was caused by immune dysregulation from his underlying plasma cell disorder, an etiogenic role for myeloma was thought to be unlikely, given the absence of elevated serum free light chains, normal SPEP, normal immunoglobulin levels, lack of plasmacytosis on bone marrow biopsy, and stable PET scan. 

We suspect that ITP in this patient resulted from immunomodulatory effects of lenalidomide therapy. Although the patient had been on stable doses of lenalidomide since his autologous bone marrow transplant, there were signs of autoimmune phenomena and increased drug toxicity in the weeks preceding his ITP diagnosis. The patient developed unexplained alopecia which peaked in severity in late March, concurrent with development of acute thrombocytopenia. Alopecia resolved upon discontinuation of lenalidomide. He also developed leukopenia, a recognized complication of lenalidomide therapy. Myelosuppression is the dose-limiting toxicity of lenalidomide therapy with grade 3/4 toxicities manifesting as neutropenia in 21%–53%, thrombocytopenia in 10–50%, and anemia in 6–9% of patients [10]. Cytopenias reverse upon dose reduction or discontinuation, as evidenced by normalization of WBC counts in our patient upon drug discontinuation. The delayed presentation of thrombocytopenia after drug discontinuation and presence of normal megakaryocyte numbers on marrow examination preclude direct drug toxicity as a mechanism for this patient's thrombocytopenia. Furthermore, the lack of circulating drug-dependent antibodies and the gradual recovery in platelet counts suggest that his thrombocytopenia was likely autoimmune in origin. As an alternative hypothesis, it is possible that lenalidomide was controlling or containing an autoimmune response and that ITP may have been exacerbated by drug discontinuation. However, this scenario is not likely given that the patient had no prior history of ITP or other autoimmune diseases.

Autoimmune thrombocytopenia has been described in association with several drugs. Both drug-dependent antibodies and autoantibodies have been described with quinidine and sulfamethoxazole therapy [11]. Autoimmune manifestations are also seen in patients treated with heavy metals [12] and IMiD therapy, including alpha interferon and thalidomide [5–7]. The mechanism by which drugs perturb immune balance and promote autoimmunity is not well understood. In the case of IMiDs, such as alpha interferon and thalidomide or thalidomide derivatives, autoimmune dysregulation may arise from effects on T cell proliferation [13]. IMiDs modulate T cell proliferation and can potentially interfere with function of Tregs, an important T cell subset that subserves a role in peripheral tolerance [14]. Lenalidomide interferes with Treg proliferation and function [15]. It is possible that latent autoreactive T cells, whether directed at platelet antigens [16] or other autoantigens, could proliferate in the setting of impaired Treg function caused by lenalidomide. Similar observations of autoimmune thrombocytopenia or *in vitro* studies of T cell autoreactivity in lenalidomide treated patients would provide experimental support of this hypothesis.

In conclusion, we report a case of autoimmune ITP in association with lenalidomide therapy. The mechanism of thrombocytopenia appears to be distinct from its well-known myelosuppressive effects and may relate to its immunomodulatory properties.

## Figures and Tables

**Table 1:** Key clinical time points and relevant studies.

	MM diagnosis (14 months prior to ITP diagnosis)	Lenalidomide discontinuation (17 days prior to ITP diagnosis)	ITP diagnosis
WBC/HGB/PLT	8.3/15.5/253	3.1/13.3/147	4.3/13.2/1

MM labs	Normal SPEP IFE: detectable free kappa light chain Serum FLC: elevated kappa light chain (91 mg/dL), elevated kappa: lambda ratio (120)	Normal SPEP IFE: no monoclonal component Serum FLC: normal	

Other labs	Serum creatinine 1.2 mg/dL	Serum creatinine 0.9 mg/dL Direct Coombs' test negative	Serum chemistries normal LDH normal TSH normal HIV negative Hepatitis B and C negative ANA 1 : 160 Anticardiolipin antibodies negative Direct Coombs' test negative Lenalidomide drug-dependent antibodies negative

Peripheral blood film			Peripheral blood film with thrombocytopenia, no schistocytes or dysplasia

Bone marrow	Normocellular bone marrow with trilineage hematopoesis, 2% plasma cells		Normocellular bone marrow with trilineage hematopoesis, adequate megakaryocytes, 2% plasma cells, normal cytogenetics

FLC: free light chains; HGB: hemoglobin; IFE: immunofixation electrophoresis; ITP: immune thrombocytopenic purpura; MM: multiple myeloma; PLT: platelet count; SPEP: serum protein electrophoresis; WBC: white blood cell count.
